# Prevalence and Risk Factors of Amblyopia among Refractive Errors in an Eastern European Population

**DOI:** 10.3390/medicina54010006

**Published:** 2018-03-20

**Authors:** Valeria Mocanu, Raluca Horhat

**Affiliations:** 1 Department of Ophthalmology, Victor Babes University of Medicine and Pharmacy, 300041 Timisoara, Romania; mocanu.valeria@umft.ro; 2 Clinic of Paediatric Surgery, Emergency Children’s Hospital Louis Turcanu, 300011 Timisoara, Romania; 3 Department of Biophysics, Victor Babes University of Medicine and Pharmacy, 300041 Timisoara, Romania

**Keywords:** amblyopia, prevalence, risk factors, refractive errors

## Abstract

*Background and objective:* Amblyopia is the leading cause of visual impairment in children and adults and is very common during childhood. The aim of this study was to identify the prevalence and the risk factors of amblyopia in a pediatric population with refractive errors from an Eastern European country. *Materials and methods:* A total of 1231 children aged 5–16 years, who had refractive errors and were examined from January to August 2017, were enrolled in a cross-sectional population-based study. Every child underwent a complete ophthalmological exam. Amblyopia was defined as a visual acuity (VA) of less than 0.63. The study respected the Multi-Ethnic Pediatric Eye Disease Study (MEPEDS) criteria for defining amblyopia (MEPEDS, 2008). Parents participated in a face-to-face interview. The questionnaire contained details about their family history of amblyopia; the child’s maternal nutritional status in the preconception period; their history of maternal smoking or work in a toxic environment; the child’s birth, and the child’s history of congenital naso-lacrimal duct obstruction (CNLDO). *Results:* Amblyopia was identified in 2.8% of the participants. The ocular conditions hyperopia (*p* = 0.0079), astigmatism (*p* = 0.046), anisometropia (*p* < 0.001), esotropia (*p* < 0.001), exotropia (*p* = 0.0195), and CNLDO (*p* < 0.001), as well as a family history of amblyopia (*p* < 0.001), were associated with amblyopia. The non-ocular risk factors for amblyopia that were found in the study included low birth weight (*p* < 0.0009), prematurity (*p* < 0.001), an Apgar score under 7 (*p* = 0.0008), maternal age, maternal smoking history or work in toxic environment (*p* < 0.001), and maternal body mass index in the preconception period (*p* < 0.003). *Conclusions:* Some of the risk factors we identified for amblyopia are modifiable factors. This is an important observation as an adequate health education program can provide the relevant information for future mothers that will allow for a better management of the condition. We also wanted to highlight the need for amblyopia screening starting from the age of 3 years in case of significant parental refractive errors, strabismus, prematurity, and maternal risk factors.

## 1. Introduction

Amblyopia, the leading cause of visual impairment in children and adults, is defined as unilateral or bilateral visual loss with no ocular pathology [1]. Amblyopia is very common during childhood, with a worldwide prevalence ranging between 0.2% and 6.2%. Risk factors in amblyopia can be divided into ocular and non-ocular risk factors. It is associated with refractive error, strabismus, or anisometropia [1,2,3,4,5]. One-third of the population will present a refractive error (myopia, hyperopia, astigmatism) or amblyopia in 2020 [6]. In total, 5.8–11.6% of the United States, Western European, and Australian population suffer from hyperopia and 16.4–26.6% from myopia [7]. The Avon Longitudinal Study of Parents and Children (ALSPAC) study from the United Kingdom identified that one in 30 children aged 7 years had amblyopia [8]. Another ocular risk factor for amblyopia was identified as congenital nasolacrimal duct obstruction (CNLDO). CNLDO is one of the most common pathologies diagnosed in a pediatric ophthalmology unit, affecting 20% of the infant population. It may lead to amblyopia due to the blurry vision secondary to constant epiphora and intermittent discharge [9].

Different studies investigating perinatal, socioeconomic, and demographic risks identified non-ocular factors associated with amblyopia such as maternal smoking during pregnancy, prematurity, Apgar score, and neonatal intensive care unit hospitalization [1,2,8,10].

There are two major criteria guidelines for amblyopia—one from the American Association for Pediatric Ophthalmology and Strabismus (AAPOS), and the other from the Multi-Ethnic Pediatric Eye Disease Study (MEPEDS) [11,12].

Amblyopia identification is an important step in ensuring the normal development of children. Recognizing and avoiding the amblyopic risk factors will decrease the vision loss in childhood and will increase the quality of life of the adult [13]. In order to identify amblyopia, a complete ophthalmological examination is mandatory. In some parts of the world, the access to medical care is difficult. For example, the Alaska Blind Child Discovery project reported that only 6% of the 3930 enrolled children had had previous ophthalmological examinations [14]. 

In the Western part of Romania, there have been no previous attempts to determine the prevalence and the risk factors for amblyopia in the pediatric population. Another particularity for this region is related to poor medical education; children in this region are referred to the ophthalmologist with significant delay.

The study had two main aims: first, to determine the prevalence of amblyopia among pediatric patients with refractive errors, and second, to analyze the presence of risk factors for amblyopia that were either previously mentioned in the literature or are identified as new risk factors in this subpopulation. 

## 2. Material and Methods

This is a cross-sectional study conducted in the Emergency Children’s Hospital Louis Turcanu, Timisoara, Romania—the only pediatric ophthalmology center in the western region of the country- between January and August 2017. From all the patients examined in this period in the Ophthalmology Department, those with refractive errors were selected. After a complete ophthalmological examination, these patients were divided into two groups: amblyopic and non-amblyopic. The amblyopic group consisted of 35 patients, while the non-amblyopic group had 1196 subjects with refractive errors. The size of the groups was tailored to fulfil the minimum requirements for a sample size calculation. Using a significance level of 0.05, a power of 0.8 and a prevalence level of 2.1%, the minimum size of the group would have been 24 patients with amblyopia and 1143 subjects without amblyopia. 

All medical procedures were performed with the approval of the Ethics Committee of the Emergency Children’s Hospital Louis Turcanu, Timisoara. All medical procedures also respected the Declaration of Helsinki and had the support of the Victor Babes University of Medicine and Pharmacy, Timisoara (AMBLYOGAIN approval 768/17.01.2017). Informed consent for the examination procedures and the use of the patients’ data was signed by the parents.

Every child included in the study underwent a complete ophthalmological examination, which included a test for visual acuity, a measurement of the refractive error after cycloplegia, an evaluation of the eye movements, a cover test, prism cover–uncover tests for near and distance, a slit-lamp, and a fundus examination. 

Every parent participated in a face-to-face interview with the medical staff. The questionnaire included details about their demographic status (rural/urban), their amblyopia family history for first degree relatives, the age, the weight, and the height of the mother in the conception period, and their history of maternal smoking or work in a toxic environment during pregnancy (history of exposure to toxic, volatile substances such as chromium VI and phthalates, which are used in shoes and plastics factories). Body Mass Index (BMI) was calculated using the weight and the height data obtained from mothers. Data concerning birth was collected from an interview with the parents and the child’s health record: weeks of gestation, weight in grams and height in centimeters at delivery, Apgar score, and type of delivery (normal vaginal birth/caesarean birth). Ocular pathology history such as CNLDO was also registered.

Monocular visual acuity (VA) was tested using a Snellen chart. The best corrected visual acuity (BCVA) was noted in decimal form in the patient’s medical chart. 

Cycloplegic refraction was measured with a Nidek AR-600 Autorefractometer (Aichi, Japan) 30 min after the instillation of the last drop from 3 of the cyclopentolate 1% spaced at 5 min. In cases of dark irides or difficult dilatation, tropicamide 1% and/or phenylephrine 2.5% were used. In order to diagnose cycloplegia, a minimum of 6 mm pupil diameters or the absence of the pupillary light reflex was considered mandatory. 

Myopia was considered in cases of a refraction of −0.50 D or less. Myopia was stratified into low (−0.50 to −2.99 D), medium (−3.00 to −5.99 D) and high (−6.00 D or greater). Hyperopia was defined as a refraction of ≥+0.5 D. Low hyperopia was considered for refractions of +0.50 D to +1.99 D, and significant hyperopia was considered for refractions of +2.00 D or more. We defined astigmatism as a cylinder of ≥−0.5 D. Low astigmatism was considered for a cylinder from −0.50 D to −1.00 D, moderate from −1.25 D to −2.50 D, and high from −2.75 D to −5.00 D [15].

Amblyopia was defined as a best corrected visual acuity (BCVA) less than 0.63 (equivalent to Snellen VA <20/32 or <0.2 log MAR unit) without the existence of an eye structure or visual pathway pathology. 

This study respected the MEPEDS criteria for defining unilateral amblyopia as a 2-line difference in BCVA between eyes with one of the following elements: (1) anisometropia (≥3.00 D anisomyopia; ≥1.00 DSE anisohyperopia; ≥1.50 D anisoastigmatism); (2) intermittent or constant strabismus; (3) a history of strabismus surgery; or (4) a presence or history of visual axis obstruction for at least 1 week (e.g., corneal opacity, ptosis, eyelid hemangioma, cataract, or aphakia). Bilateral amblyopia was defined as bilaterally decreased BCVA associated with significant ametropia (≥6.00 D myopia, ≥4.00 D hyperopia, ≥2.50 D astigmatism) or a history of bilateral visual axis obstruction. Anisometropia was defined as a difference between the patient’s eyes of ≥1.00 diopters (D) for hyperopia, ≤−3.00 D for myopia, and ≥1.50 D for astigmatism [12]. 

To identify strabismus, prism cover–uncover tests for near (33 cm) and distance (6 m) were performed. A deviation of ≤10 prism diopters was defined as microstrabismus. Constant strabismus was diagnosed if tropia was present at distance and near fixation; if it was not present, then it was defined as intermittent. Strabismus was classified by its tropia primary direction as esotropia, exotropia, and vertical. 

Preterm birth was defined as less than 37 weeks in gestational age. Low birth weight was defined as a birth weight under 2500 g. Delivery was classified as either a normal vaginal birth or a caesarean birth.

The Apgar score is an index of the health status of the baby in the first 5 min after birth, and it is defined as an analysis of 5 elements: skin color, heart rate, reflex irritability grimace, muscle tone, and respiratory effort [9,10]. An Apgar score of 7–10 is considered to be generally normal, 4–6 is considered to be fairly low, and ≤3 is considered to be critically low [16]. 

The BMI or the Quetelet index was calculated using the following formula: the weight in kilograms divided by the square of the height in meters. An underweight status was defined as a BMI <18.5, while an overweight status was defined as a BMI >25 [17].

### Statistical Analysis

Statistical analysis consisted of a descriptive part in which the mean and standard deviations were computed. The analysis continued with checking the normality of the data distribution using Shapiro–Wilk’s test. The lowest probability obtained was *p* = 0.11, which allowed us to assume normality of the data distribution. This was followed by testing the statistical differences between the 2 study groups using an unpaired *t*-test, a Mann–Whitney test and a chi-squared test based on the type of variable. The next step was a risk analysis which allowed the calculation of odds ratios (OR). All of these were performed using Epi Info software package version 7.2 (https://www.cdc.gov/epiinfo/index.html). Statistical analysis concluded with a logistic regression performed in R version 3.4.1 (https://www.r-project.org/) in order to quantify the impact of all the risk factors described above. The binary outcome modelled was the presence or absence of amblyopia. The model included a total of 18 potential relevant risk factors. These potential risk factors were age (V2), presence of NLDO (V3), provenance (V4), family history (V5), mother’s age (V6), mother’s nutritional status (V7), toxic exposure and smoking (V8), associated pathology during pregnancy (V9), low birth weight (V10), gestational age (V11), associated pathology at birth (V12), birth length (V13), APGAR score (V14), caesarean (V15), anisometropia (V16), esotropia (V17), exotropia (V18), and the severity of the uncorrected VA (V19). 

## 3. Results

In total, 1231 subjects with refractive errors were included in the study, of which 35 (2.8%) were diagnosed with amblyopia. Of the 35 participants with amblyopia, 24 (68.8%) were girls. In the non-amblyopic group, 792 (66.22%) were girls and 404 (33.77%) were boys. No statistical significance was identified in sex difference (*p* = 0.70). The mean age in the amblyopic patients was 9.94 (2.75, range 5–16 years) and 10.1 (2.5, range 5–18 years) in the non-amblyopic children (*p* = 0.306). 

The mean BCVA in the amblyopic group was 0.349 (0.199, range 0.06 to 0.63). Unilateral amblyopia was diagnosed in 18 (51.4%) cases. Amblyopia was found in the right eye in 7 (39%) children and in the left eye in 11 (61.1%) children. Bilateral amblyopia was identified in 17 (48.6%) cases due to anisometropia. Amblyopia in the better eye was caused by high refractive error.

In Table 1, Table 2 and Table 3, we can observe ocular conditions that are associated with amblyopia such as hyperopia, astigmatism, and anisometropia. The association between anisometropia and amblyopia was highly statistically significant (*p* < 0.001).

As per the above table, all the analyzed ocular conditions associated with amblyopia were found to be statistically significant risk factors. 

In regards to the non-ocular conditions associated with amblyopia, their statistical significance as risk factors for amblyopia was analyzed in Table 4. With the exception of gender and caesarian birth, all the other non-ocular conditions analyzed were statistically significant risk factors. 

In the logistic regression model, 14 factors were statistically significant (*p*-value < 0.05), with one at the limit of statistical significance (V15). These risk factors were age (V2), NLDO (V3), family history (V5), mother’s age (V6), mother’s nutritional status (V7), toxic exposure and smoking (V8), low birth weight (V10), gestational age (V11), Apgar score (V14), caesarian birth (V15), anisometropia (V16), esotropia (V17), exotropia (V18), and severity of uncorrected VA (V19). Out of these 14 factors, four had a negative contribution towards the occurrence of the amblyopia, in that their increase reduced the risk of amblyopia (V2, V11, V14, V15). The logistic regression equation is as follows:

Probability of amblyopia = 1/(1 + exp(−(−0.4993*V2 + 1.8852*V3 + 3.8451*V5 + 0.2696*V6 + 0.5226*V7 + 5.4376*V8 + 5.8511*V10 − 3.9024*V11 − 1.0813*V14 − 2.4034*V15 + 4.6349*V16 + 7.9040*V17 + 2.7428*V18 + 4.2038*V19 − 0.6794))).

The full details can be found in the Supplementary Table. The merit of this logistic regression model is that it allows for the selection of all the relevant predictors on the one hand, and on the other hand, it allows for the assessment of the added effect of all of these relevant predictors.

## 4. Discussion

The first aim of this study was to determine the prevalence of amblyopia in a subpopulation of pediatric patients with refractive errors. In our study, which included 1231 participants, the prevalence of amblyopia was 2.8%. Major studies concerning the prevalence of amblyopia were performed in the general population. Due to the fact that the reference population in our study was different to the general population, we expected our prevalence to be different too. 

In addition, there is a significant variability in the prevalence that is reported by large studies. Different values of prevalence were obtained due to the VA criteria and the age of the examined population. Studies that exclude patients with a VA higher than 0.5 (equivalent Snellen 20/40, 0.3 log MAR) obtain a lower prevalence of amblyopia. In contrast, there are studies with no VA criteria such as the study from the Blue Mountains area west of Sydney. In this study, the prevalence of amblyopia was 3.9% [18]. In general, the prevalence of amblyopia in school children varies from 0.2% in Indonesia to 6.7% in Chile [19,20].

Major studies analyzing the prevalence of amblyopia are briefly described in Table 5.

The second aim of the study was to identify risk factors for amblyopia in a subpopulation with refractive errors. In the medical literature, we were able to find studies concerning the risk factors for amblyopia in the general population and studies analyzing the risk factors for refractive errors. For this reason, our results cannot be completely compared with previous articles.

From the ocular risk factors, we found an association of amblyopia with refractive errors such as hyperopia and astigmatism, with anisometropia and strabismus. 

While analyzing the values of the refractive errors, we observed a statistically significant difference for hyperopia and astigmatism (*p* = 0.0079, *p* = 0.046). These refractive errors were also identified as risk factors for amblyopia in the general population. In the Australian preschool children study, hyperopia, defined as ≥+2.00 D, was identified in 18 of the 27 amblyopic preschool children (66.7%). Myopia, defined as ≥−0.50 D, was present in two (7.4%) of the 27 amblyopic children. In 13 (48.1%) of the 27 amblyopic cases, astigmatism (≥1.00 D) was measured. Six (22.2%) of these cases presented with significant astigmatism [1]. A longitudinal study in Tennessee, which included 221,720 children with a mean age of 39.6 months, identified 149 participants with significant hyperopia [23]. In the Australian 6-year-old children study, 58.7% presented with significant hyperopia and 8.7% presented with myopia [2].

Anisometropia was found in 10 (28.6) of the amblyopic children and was associated with amblyopia. A similar association was identified in the Singapore study (*p* < 0.001; OR 20.6, 95% CI 4.6–91.7), the Sydney Paediatric Eye Disease Study (SPEDS) (OR 27.8, 95% CI 11.2–69.3), the Sidney Myopia Study (OR 156, 95% CI 64–382), and the Australian study (OR, 27.82; 95% CI, 11.17–69.31) [1,2,22,24].

In regards to strabismus, we identified that exotropia and esotropia were risk factors for amblyopia. In the Australian preschool children study, strabismus was diagnosed in 10 of the 27 (37.0%) amblyopic children; five (18.5%) were diagnosed with esotropia and four (14.8%) were diagnosed with exotropia [1]. The study that investigated 6-year-old amblyopic children identified esotropia in 11 of the 18 cases (61.1%) and exotropia in three of the 18 cases (16.7%) [2]. Similar proportions of exotropia and esotropia were obtained in the Friedman study which included white preschool children [4]. In the East Asian children study, esotropia was less prevalent than exotropia, probably due to the fact that the Asian population is more susceptible to myopia than to hyperopic refractive errors [5,25,26,27,28,29,30]. In contrast, an equal proportion of esotropia and exotropia was observed in East Asian children from the SPEDS [1]. In a Singapore study that included 1682 young children, exotropia in the amblyopic group was found in two cases and esotropia in one. The study found an association between amblyopia and strabismus (*p* = 0.001; OR 18.0, 95% CI 3.3–97.8) [22]. Other studies such as the Sydney Myopia Study (SMS) and the SPEDS also reported strabismus as a risk factor for amblyopia (OR 13.1, 95% CI 4.2–40.3 and OR 65, 95% CI 30–144) [1,2,24].

A family history of amblyopia was another risk factor identified by our study, which is consistent with medical literature. The ALSPAC (272 amblyopic/7825, 7 years old) reported that first degree relatives with amblyopia represent a risk factor in developing amblyopia [8]. Chia et al., in their Singaporean study, observed that in some sibling cases, both of the children were affected by amblyopia (2.3%) [22]. 

The present study found that CNLDO was highly associated with amblyopia. We did not find similar results in the literature. Other studies analyzed the presence of amblyopia among children with CNLDO. A study investigating 210 children with CNLDO identified an association between CNLDO and amblyopia in 3.9% of cases [9]. Matta and Silbert observed that 22% of the children aged under 3 years old with CNLDO presented risk factors for amblyopia [31]. Simon et al. identified five children with anisometropic amblyopia and CNLDO [32]. Chalmers and Griffiths evaluated 130 children with unilateral CNLDO. Amblyopia was present in five of these cases [33]. Similar results were obtained by Bagheri et al. [34]. Ellis et al. studied the influence of tear film disturbance on visual maturation but found no association [9].

We found that the non-ocular risk factors for amblyopia in a pediatric population with refractive errors were low birth weight, prematurity, an Apgar score under 7, maternal age, maternal smoking history or work in toxic environment, and maternal BMI in the preconception period. 

Similar associations have been analyzed in different studies in the general population. The 6-year-old Sydney school children study reported a significant association between amblyopia and preterm birth (*p* < 0.001) and low birth weight (*p* = 0.03) [2,24,35,36]. Amblyopia was diagnosed in 31% of preterm births. In children born at less than 37 weeks of gestation, a 5-fold greater risk for amblyopia was identified (OR, 5.4; 95% CI, 2.3–12.3). A similar result was obtained for a birth weight less than 2500 g, where these children present a 5-fold greater risk for amblyopia (OR, 4.8; 95% CI, 1.9–11.8) [2]. The SPEDS identified a significant association between preterm birth (<37 weeks; OR, 3.23; 95% CI, 1.35–7.71; *p* = 0.008) and low birth weight (<2500 g; OR, 4.71; 95% CI, 1.83–12.13; *p* = 0.001) [1].

A study from the United Kingdom, which included 293 prematurely born children, reported that low birth weight and gestational age are risk factors for amblyopia [37]. An Iranian study that included 164 children with refractive errors, 73 amblyopic children, and 91 non-amblyopic children, found that preterm birth presents a 7-fold greater risk for amblyopia (OR, 7.11; 95% CI 2.28–22.14). Children with a low birth weight had a 6-fold greater risk for amblyopia (OR, 6.49; 95% CI 2.29–18.32) [38].

In contrast, the Singapore study found no association between amblyopia and prematurity [22]. In the Australian preschool children study, amblyopia was not associated with low birth weight <2500 g (OR, 2.61; 95% CI 0.33–20.87), with prematurity <37 weeks (OR, 1.81; 95% CI 0.37–8.75), or with a maternal history of smoking during pregnancy (OR, 1.41; 95% CI 0.26–7.69) [1]. 

In addition, we found that the type of delivery had no association with amblyopia. Similar results were obtained by an Iranian study with a refractive error population, where 9 (12%) of the amblyopic children were conceived through a caesarean birth, and no association with amblyopia was identified (OR, 1.29; 95% CI, 0.52–3.18) [38].

We identified only one study analyzing the association between Apgar score and amblyopia in the general population. It included 5834 preschool children from the Yuhuatai District, Nanjing, China, and it observed that amblyopia was associated with an Apgar score under 7 (OR, 1.65, 95% CI 1.03–2.55) [39]. 

As for the maternal factors, we found that smoking during pregnancy, work in a toxic environment, and a maternal age over 35 are risk factors for amblyopia in a pediatric population with refractive errors. 

Nicotinic acetylcholine receptors appear to play an important role in refractive development [40,41]. The nicotine present in the cigarettes activates the nicotinic acetylcholine receptors [42]. The activation of nicotinic acetylcholine receptors antagonizes the muscarinic acetylcholine receptors involved in the elongation of the eye [43]. Some studies identified that a toxic pre-natal environment may lead to disturbances in the neural growth of the cones and in optic disc hypoplasia due to the reduction of placental flood flow [44]. Lempert reported that hypoplasia of the optic nerve is associated with amblyopia [45]. 

The association between maternal smoking and amblyopia in the general population is confirmed by various studies. The ALSPAC study from 2008 identified an association between amblyopia and maternal smoking (OR 1.4, 95% CI 1.0–1.9) [22]. The Australian 6-year-old children study obtained a borderline association between maternal smoking during pregnancy and amblyopia. In total, 22.6% of the children who were conceived by mothers who smoked during pregnancy presented amblyopia, while 11.7% were non-amblyopic [2]. In countries such as Chile, Denmark, or the United States, a high prevalence of smoking women was reported (28% to 58%) [45,46]. In these countries, an increased prevalence of amblyopia was observed: 6.7% in Chile, 2.9% in Denmark, and 5% in the United States [19,45,47]. In contrast, countries with a low prevalence of smoking women (e.g., China (1.7%), Indonesia (2.6%), Saudi Arabia (1.3%), and Singapore (4%)) reported a lower rate of amblyopia (0.72%–1.43%) [20,21,45]. A Japanese study found no association between parental smoking and a visual acuity less than 0.7 [48]. 

Maternal smoking was also found as a risk factor for amblyopia in populations with refractive errors. The meta-analysis of Li et al. included 3282 children with refractive errors and 318 with amblyopia. Children from smoking mothers had a 1.47-fold higher risk for amblyopia than those whose mothers did not smoke during pregnancy. The results were statistically significant for amblyopia (95% CI 1.12–1.93). [13]. 

A maternal age over 35 was another risk factor identified by our study. The Collaborative Prenatal Project of the National Institute of Neurological Disorders and Stroke (CPP) compared maternal ages between 30–34 years of age with maternal ages between 20–24 years of age, and they reported that an older age at preconception is a risk factor for esotropia (OR 1.4, 95% CI 1.1–1.7) [49]. SMS identified no association between increased maternal age and amblyopia [24].

We also analyzed the BMI of mothers at preconception and we found that being underweight or being overweight are risk factors for amblyopia. We found no data to compare with in the medical literature.

We acknowledge that our study has several limitations. First of all, the reference population consists of a pediatric population with refractive errors and not the general population as is the case for the majority of the studies from the literature. Secondly, our study had a small number of participants compared to large studies. Thirdly, our results did not take socioeconomic factors into account, such as social class or diet. 

The mean age of the study population was 9.94 (2.75) so the results from the visual acuity measurements were certain. However, the data obtained from the parents (e.g., maternal smoking history, family history of amblyopia, weight of the mother at preconception) may be questionable.

We concluded our statistical analysis with a logistical regression model. Due to the fact that the study comprises a subpopulation of pediatric patients with refractive errors, we are not expecting the equation to be applicable to the general population. However, it can serve as a starting point for further research in the field of amblyopia and refractive errors. 

## 5. Conclusions

This is the first study investigating the prevalence of amblyopia in a pediatric population with refractive errors from the Western region of Romania. The value found was 2.8%. Some of the risk factors we identified for amblyopia, such as maternal smoking during pregnancy, working in a toxic environment, low maternal BMI in preconception, and untreated CNLDO, are modifiable factors. This is an important observation as an adequate health education program can provide the relevant information for future mothers that will allow for a better management of the condition.

Through this study, we wanted to highlight the need for amblyopia screening. Every child should benefit from an ophthalmological examination starting from the age of 3 years in case of significant parental refractive errors, strabismus, prematurity, and maternal risk factors. An option for children with no history of prematurity or obvious ocular pathology would be a screening of their visual acuity at the age of 6 years when they start school.

## Figures and Tables

**Table 1:** Refractive errors in the amblyopic and non-amblyopic group.

Refractive Errors	Amblyopic Group No. (%)	Non-Amblyopic Group No. (%)
***Myopia*** Low myopia (−0.50 D to −2.99 D) Medium myopia (−3.00 D to −5.99 D) High myopia (≥−6.00 D)	4 (11) 1 (25) 2 (50) 1 (25)	456 (38.12) 416 (91.22) 40 (8.8) 0 (0)
***Hyperopia*** Low hyperopia (+0.50 D to +1.99 D) Significant hyperopia ≥+2.00 D	13 (37.1) 2 (5.7) 11 (84.6)	520 (43.47) 252 (48.46) 268 (51.55)
***Astigmatism*** Low astigmatism (−0.50 D to −1.00 D) Moderate astigmatism (−1.25 to −2.50 D) High astigmatism (−2.75 D to −5.00 D)	18 (51.4) 4 (22) 8 (44) 6 (33)	220 (18.39) 152 (69.09) 60 (27.2) 8 (4)

**Table 2:** Value of the refractive errors in the two study groups.

Refractive Errors	Amblyopic Group		Non-Amblyopic Group	
	Mean Value (SD)	Range	Mean Value (SD)	Range
Myopia, D	−4 (2.524)	−7.75 to −2.25	−1.717 (0.829)	−4 to −0.5
Hyperopia, D	3.75 (1.895)	1.5 to 8.5	2.078 (0.881)	0.75 to 5.25
Astigmatism, D	2.513 (1.525)	0.75 to 5	1.339 (0.476)	0.75 to 2.75

**Table 3:** Ocular conditions associated with amblyopia.

Risk Factor	Amblyopic Children No. (%)	Non-Amblyopic Children No. (%)	*p*	Odds Ratio (95% CI)
Anisometropia	10 (28.6)	4 (0.3)	<0.001	119.20 (35.00–405.91)
Esotropia	20 (57.1)	136 (11.37)	<0.001	10.39 (5.20–20.78)
Exotropia	4 (11)	10 (0.8)	0.0195	3.38 (1.14–9.99)
Family history	6 (17)	20 (1.6)	<0.001	12.17 (4.55–32.54)
CNLDO	14 (40)	160 (13.37)	0.000008	4.32 (2.15–8.66)

**Table 4:** Non-ocular conditions associated with amblyopia.

Risk Factor	Amblyopic Children No. (%)	Non-Amblyopic Children No. (%)	*p*	Odds Ratio (95% CI)
Demographic factor (urban/rural)	10 (28.6)	604 (50.50)	0.0105	2.55 (1.21–5.36)
Gender (female/male)	24 (68.6)	792 (66.22)	0.771	1.11 (0.54–2.29)
Maternal BMI <18.5	19 (54.3)	164 (13.71)	<0.001	7.47 (3.77–14.83)
Maternal BMI >25	3 (9)	20 (1.7)	0.002	5.51 (1.56–19.50)
Maternal age ≥35 years	3 (9)	12 (1)	0.000057	9.25 (2.49–34.39)
Toxic environment	11 (31.4)	24 (2)	<0.001	22.38 (9.86–50.83)
Smoking during pregnancy	17 (48.6)	156 (13.04)	<0.001	6.30 (3.18–12.48)
Caesarean birth	18 (51.4)	528 (44.19)	0.392	1.34 (0.68–2.62)
Prematurity	14 (40)	152 (12.7)	<0.001	4.58 (2.28–9.20)
Apgar score <7	4 (11)	28 (2.3)	0.0008	5.38 (1.78–16.28)
Birth weight <2500 g	11 (31.4)	148 (12.37)	0.0009	3.25 (1.56–6.76)

**Table 5:** Prevalence of amblyopia in the pediatric general population in the listed studies.

Study	Age of Patients	Number of Patients	Prevalence (%)	References
Sydney Paediatric Eye Disease Study	6–72 months	1422	1.9	Pai et al. (2012) [1]
ALSPAC Study	7 years	7825	3.6	Wiliams et al. (2008) [8]
Baltimore Pediatric Eye Disease Study	30–72 months	2546	08–1.8	Friedman et al. (2009) [4]
Singaporean Chinese Children Study	30–72 months	1682	1.19	Chia et al. (2010) [5]
La Florida, Chile Study	5–15 years	5303	6.5	Maul et al. (2000) [19]
Saudi Arabia Study	3–6 years	102	1.3	Bardisi and Bin Sadiq (2002) [21]
Strabismus, Amblyopia and Refractive Error in Singaporean Preschoolers Study (STARS)	30–72 months	3009	0.8	Chia et al. (2013) [22]

## Supplementary Materials

The following are available online at http://www.mdpi.com/1010-660X/54/1/6/s1, Table S1: Logistic regression model.
