# Supplementary material for: Prevalence and Risk Factors of Amblyopia among Refractive Errors in an Eastern European Population
Source: Medicina (Kaunas). 2018 Mar 20;54(1):6. doi: 10.3390/medicina54010006 (PMC6037249; doi:10.3390/medicina54010006)
Supplement: Supplementary file 1 [file medicina-54-00006-s001.pdf]

Supplementary Table. Logistic regression model.

Index of risk factor	Name of the risk factor	OR	CI	p-value
V2	Age	0.61	0.42- 0.82	0.003
V3	NLDO	6.59	1.26- 47.4	0.037
V5	Family history	46.77	4.24- 662.38	0.002
V6	Mother's age	1.31	1.06- 1.72	0.026
V7	Mother's nutritional status	12.46	1.73- 118.97	0.016
V8	Toxic exposure and smoking	229.9	17.86- 5611.48	<0.001
V10	Low birthweight	347.62	9.51- 40383.53	0.006
V11	Gestational age	0.02	0.0004- 0.4	0.023
V14	APGAR score	0.34	0.16- 0.71	0.002
V15	Caesarean	0.09	0.005- 0.77	0.058
V16	Anisometropia	103.01	14.77- 1046.93	<0.001
V17	Esotropia	2707.96	200.03- 83807.39	<0.001
V18	Exotropia	15.53	2.8- 124.33	0.004
V19	Severity of uncorrected AV	66.94	3.77- 1203.59	0.002
